# Cathelicidin (LL‐37) causes expression of inflammatory factors in coronary artery endothelial cells of Kawasaki disease by activating TLR4–NF‐κB–NLRP3 signaling

**DOI:** 10.1002/iid3.1032

**Published:** 2023-09-26

**Authors:** Feifei Si, Yaheng Lu, Yizhou Wen, Tingting Chen, Yingzi Zhang, Yanfeng Yang

**Affiliations:** ^1^ Pediatric Cardiovascular Department, Chengdu Women's and Children's Central Hospital, School of Medicine University of Electronic Science and Technology of China Chengdu China

**Keywords:** inflammatory response, Kawasaki disease, LL‐37, NLRP3, toll‐like receptors 4

## Abstract

**Background:**

Kawasaki disease (KD) is a type of vasculitis with an unidentified etiology. Cathelicidin (LL‐37) may be involved in the development of the KD process; therefore, further research to investigate the molecular mechanism of LL‐37 involvement in KD is warranted.

**Methods:**

Enzyme‐linked immunosorbent assay (ELISA) was used to detect the levels of tumor necrosis factor‐α (TNF‐α), interleukin (IL)‐1β, NLRP3, and LL‐37 in the sera of healthy subjects, children with KD, and children with pneumonia. Subsequently, human recombinant LL‐37 or/and toll‐like receptors 4 (TLR4)‐specific inhibitor TAK‐242 stimulated human coronary artery endothelial cells (HCAECs), CCK‐8 was used to detect cell proliferation, flow cytometry to detect apoptosis, transmission electron microscopy to observe cytoskeletal changes, Transwell to measure cell migration ability, ELISA to detect inflammatory factor levels, Western blot analysis to analyze protein levels of toll‐like receptors 4 (TLR4) and NF‐κB p‐65, and quantitative real‐time polymerase chain reaction (qRT‐PCR) to determine LL‐37, NLRP3 mRNA levels.

**Results:**

In this study, we found that the level of LL‐37 was highly expressed in the serum of children with KD, and after LL‐37 stimulation, apoptosis was significantly increased in HCAECs, and the expression levels of TLR4, NLRP3 and inflammatory factors in cells were significantly enhanced. Intervention with the TLR4‐specific inhibitor TAK‐242 significantly alleviated the LL‐37 effects on cellular inflammation, TLR4, NLRP3 promotion effect.

**Conclusions:**

Our data suggest that LL‐37 induces an inflammatory response in KD coronary endothelial cells via TLR4‐NF‐κB‐NLRP3, providing a potential target for the treatment of KD.

## Graphical abstract

We have demonstrated for the first time that evaluated the associations between Cathelicidin LL‐37 and Kawasaki disease (KD) in Chinese children. The level of LL‐37 was highly expressed in the serum of children with KD. Intervention with the toll‐like receptors 4 (TLR4)‐specific inhibitor TAK‐242 significantly alleviated the LL‐37 effects on cellular inflammation, TLR4, NLRP3 promotion effect.

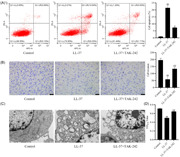

## 1 INTRODUCTION

Kawasaki disease (KD) is an acute systemic inflammatory syndrome of small and medium‐sized blood vessels that commonly affecting children under 5 years old, with most of cases occurring in children between the ages of 1 and 2 years, and is now the leading cause of acquired heart disease in children in developed countries.^1^ The danger of KD lies in coronary artery damage. Nearly 20% of children with KD can have coronary artery dilatation, and in serious cases, coronary artery aneurysm, stenosis, or even thrombosis, which can cause myocardial ischemia or even myocardial infarction, and is associated with sudden death in young adults, therefore, KD is also called “coronary heart disease” in children.^2^, ^3^ Sadly, vasculitis and even coronary aneurysms are not the endpoint of KD, which has been shown to be a high‐risk factor for coronary atherosclerosis in adults.^4^, ^5^ However, the etiology and mechanism of KD remain unclear. Therefore, it is important to study the mechanism of KD and its coronary artery injury.

Cathelicidin (LL‐37) is the only member of the family of antimicrobial peptides cathelicidins in the human body and is an important component of the natural immune system of the organism.^6^ LL‐37 is widely present in neutrophils, mononuclear macrophages, epithelial cells, and endothelial cells.^7^ LL‐37 is currently thought to be closely associated with tumors, bacterial sepsis, and adult coronary artery disease.^8^, ^9^ Together with other antimicrobial substances in the body, LL‐37 forms the first chemical line of defense of the host against microorganisms. When the organism is stimulated by the outside world, it can release active LL‐37, which in turn causes a series of waterfall inflammatory responses.^10^ Studies have shown that LL‐37, as an important molecule of natural immunity with certain chemotactic activity, can cause neutrophils, monocytes, and T lymphocytes in circulating blood to accumulate toward the site of inflammation, and can lead to endothelial dysfunction, release a series of inflammatory factors, induce a vascular inflammatory response, induce vascular smooth muscle apoptosis, and promote the formation of coronary atherosclerosis.^11^ As a “coronary heart disease” in children, the pathogenesis of KD is similar to that of adult coronary heart disease,^12^ so the study of LL‐37 provides a new direction for our research on the etiology and prevention of coronary artery injury in KD.

The signaling mechanisms associated with LL‐37 are still under investigation. Toll‐like receptors (TLRs) are a family of receptors that mediate natural immunity, and studies have revealed that LL‐37 neutralizes lipopolysaccharide, which leads to TLR imbalance, such as activation of TLR3 and inhibition of TLR4.^13^ The expression of TLR4 was found to be significantly elevated in KD,^14^ and TLR4 can bind to ligands and then activate downstream signaling pathways such as NK‐κB and MAPK, thereby triggering a waterfall inflammatory response. Among them, the NF‐κB signaling pathway can regulate the production of inflammatory factors, cell surface receptors, transcription factors, adhesion factors, and so on, and these stimuli are associated with the development of microcirculatory disorders and vascular injury.^15^, ^16^ It is also noteworthy that LL‐37 can also induce septic shock by activating NLRP3 inflammatory vesicles that contribute to the production of a series of inflammatory factors such as interleukin (IL)‐1β.^17^ NLRP3 can interact with TLR and be recently found to slow the progression of coronary atherosclerosis in the absence of NLRP3 inflammatory vesicles.^18^ Also, NLRP3 was found to be significantly increased in the KD mouse model.^19^

Taken together, LL‐37 may be involved in the development of KD, and its abnormalities may activate the TLR4‐NF‐κB signaling pathway and NLRP3, thus triggering downstream inflammatory factors and consequently damaging vascular endothelial cells, ultimately leading to alterations in KD pathology, especially the development of coronary artery injury. Thus, this study intends to clarify the difference in LL‐37 expression in the serum of KD and normal pediatric patients, and to observe the alteration of inflammatory factors, signaling pathways, and endothelial cell function by exogenous LL‐37 and/or TLR4 inhibitor intervention. To clarify the molecular mechanism of LL‐37‐induced waterfall inflammatory response leading to the development of KD, thus providing a new strategy for the prevention and treatment of KD coronary artery injury.

## 2 MATERIALS AND METHODS

### 2.1 Collection of blood samples

Blood samples were collected from healthy physical examiners (*n* = 27, no underlying cardiac disease, no infectious disease, normal liver, and kidney function), children with KD (KD, *n* = 33, all diagnostic criteria met the criteria established by the American Heart Association in 2017,^20^ the acute phase of KD, all within 1 week of onset, before intravenous immunoglobulin (IVIG) treatment, and without any treatment, excluding any history of immunodeficiency), and pneumonia (*n* = 33, Children with acute bronchopneumonia with diagnostic criteria consistent with the diagnosis of mild bronchopneumonia in Practical Pediatrics (8th edition), without comorbid acute and chronic diseases of other systems, and without antimicrobial therapy). These subjects went to the Chengdu Women's and Children's Center Hospital between March 2020 to October 2022. There were no statistical differences in age and gender between the healthy physical examiners, KD, and pneumonia groups. Pediatric early morning fasting peripheral venous blood was collected, and serum was stored at −80°C after collection until later use. All the participants signed consent forms for participation in the academic research.

### 2.2 Cell culture and treatment

Human coronary artery endothelial cells (HCAECs, Procell) were inoculated at a cell density of 1 × 10^6^/mL in RPMI 1640 medium (Hyclone) containing 10% fetal bovine serum (FBS, Viva Cell). The cells were incubated at 37°C in a 5% CO_2_ incubator. HCAECs cells of logarithmic growth phase were taken and placed in 6‐well plates (Biolgix), and these endothelial cells were respectively treated with 2, 5, 10, and 15 μmol/L of human recombinant LL‐37 (MCE), in addition, HCAECs will be treated with optimal concentrations of LL‐37 and 100 μg/L of TLR4 inhibitor (TAK‐242, MCE) and cultured for 24 h for subsequent analysis.

### 2.3 Cell proliferation

The above cells were cultured for 24 h. Ten microliters of CCK‐8 solution was added to each well, and the incubation was continued for 2 h. The absorbance (A) was measured at 450 nm using an enzyme marker (SpectraMax PLUS 384, Molecular Devices), and the mean value of each concentration group was calculated using four replicate wells of A per group.

### 2.4 Cell apoptosis assay

HCAECs cultured with different treatments were digested with trypsin, collected and prepared as single cell suspensions, and washed three times with prechilled PBS. The cells were then stained with 5 μL of Annexin V‐APC (KeyGEN BioTECH) and 5 μL of V‐PE (KeyGEN BioTECH) in the dark at room temperature. Subsequently, 10 μL of propidium iodide (PI, KeyGEN BioTECH) was added and immediately analyzed by flow cytometry (cytoflex, Beckman).

### 2.5 Transmission electron microscopy

HCAECs cells were prefixed with 3% glutaraldehyde (Sangon Biotech), then postfixed with 1% osmium tetroxide (Sangon Biotech), dehydrated in series acetone, and embedded in Epox812 (SPI). The semithin sections were stained with methylene blue and ultrathin sections were cut with diamond knife, stained with uranyl acetate and lead citrate (SPI). Sections were examined with JEM‐1400‐FLASH Transmission Electron Microscope.

### 2.6 Transwell assay

Prechilled at 4°C with 1:5 dilution of Matrigel was added to the Transwell upper chamber, spread well, and dried at 37°C for 70 min, five replicate wells were set up for each group. The cell concentration was adjusted to 5 × 10^5^ cells/mL, 200 μL of cell suspension was added to the upper chamber, and 600 μL of medium containing 20% FBS was added to the lower chamber. The chambers were incubated in 5% CO_2_ at 37°C for 24 h. The untransferred cells in the upper chamber were wiped off with cotton swabs, rinsed with PBS, fixed in methanol, stained with 0.1% crystal violet (BOMEI, Hefei), and then five fields of view per well were selected and photographed under a light microscope (LEICA), and the number of migrated cells in each group was counted.

### 2.7 Enzyme‐linked immunosorbent assay (ELISA)

Serum and cell samples were taken and the levels of tumor necrosis factor‐α (TNF‐α), IL‐1β, NLRP3, and LL‐37 in serum were determined according to the kit instructions (Proteintech). The kit was also used to determine the levels of inflammatory factors IL‐6, IL‐1β, IL‐17A, and TNF‐α; chemokines (CXCL1, CXCL2, CCL5, and MCP‐1), adhesion molecules (ICAM‐1, VCAM‐1, and ELAM‐1), and NLRP3 in cells. All experimental operations were performed strictly according to the manufacturer's instructions, and each sample was repeated three times.

### 2.8 Quantitative reverse transcription‐polymerase chain reaction (qRT‐PCR)

Cells were lysed for total RNA extraction using TRIzol kits (Invitrogen) according to the manufacturer's instructions. Total RNA from each sample was reverse‐transcribed into first‐strand cDNA for qRT‐PCR analysis. Gene expression levels were quantified using Takara TB Green™ PreMix Ex Taq™ (RR820A, Takara), and GAPDH as internal reference genes respectively. qRT‐PCR reaction conditions: initial denaturation at 95°C for 10 min, followed by denaturation at 95°C for 10 s, annealing at 60°C for 10 s, extension at 72°C for 10 s, 45 cycles, recording of CT values, relative expression levels were analyzed using 2^−∆∆CT^, and the experiment was repeated three times. Primer sequences were designed and synthesized by Shanghai Shenggong Bioengineering Technology Service Co., Ltd. Primer sequence: LL‐37: Sense: 5′‐GATTGTGACTTCAAGAAGGACG‐3′, Antisense: 5′‐GCAGGGCAAATCTCTT GTTATC−3′; NLRP3: Sense: 5′‐AGGACCTGGAGGATGTGGAC−3′, Antisense: 5′‐TCCAC ATGGTCTGCCTTCTCT‐3′; GAPDH: Sense: 5′‐TGACTTCAACAGCGACACCCA‐3′, Antisense: 5′‐CACCCTGTTGCTGTAGCCAAA‐3′.

### 2.9 Western blot analysis

Total cellular proteins were extracted using RIPA lysate (Servicebio) and protein concentrations were detected using BCA (Beyotime). Proteins were separated by 10% SDS‐PAGE electrophoresis and transferred to PVDF membranes, which were incubated with TBST containing 5% skim milk (Sigma) at room temperature for 2 h. The membranes were incubated overnight at 4°C with the following primary antibodies (1:2000 rabbit anti‐TLR4; 1:8000 rabbit anti‐NF‐kB p65; 1:1000 rabbit polyclonal anti‐β‐actin; Abclonal), washed with TBST, incubated with goat‐anti‐rabbit IgG (H + L)‐HRP (Affinity) for 2 h at room temperature, added ECL luminescent solution (Sigma) for color development and photographed in an autoexposure meter. The grayscale value of each protein band was analyzed by ImageProPlus software and the relative protein expression was calculated.

### 2.10 Statistical analysis

Data were statistically analyzed using SPSS 22.0 statistical software (IBM Corp), and datas were expressed as mean ± standard error of the mean (SEM), and LSD‐*t* test was used to analyze the data with only two groups, and one‐way analysis of variance (ANOVA) of variance was used to analyze the differences among multiple groups, with *p* < .05 indicating that the differences were statistically significant.

## 3 RESULTS

### 3.1 Levels of TNF‐α, IL‐1β, NLRP3, and LL‐37 in serum of patients with KD

**Figure 1:**
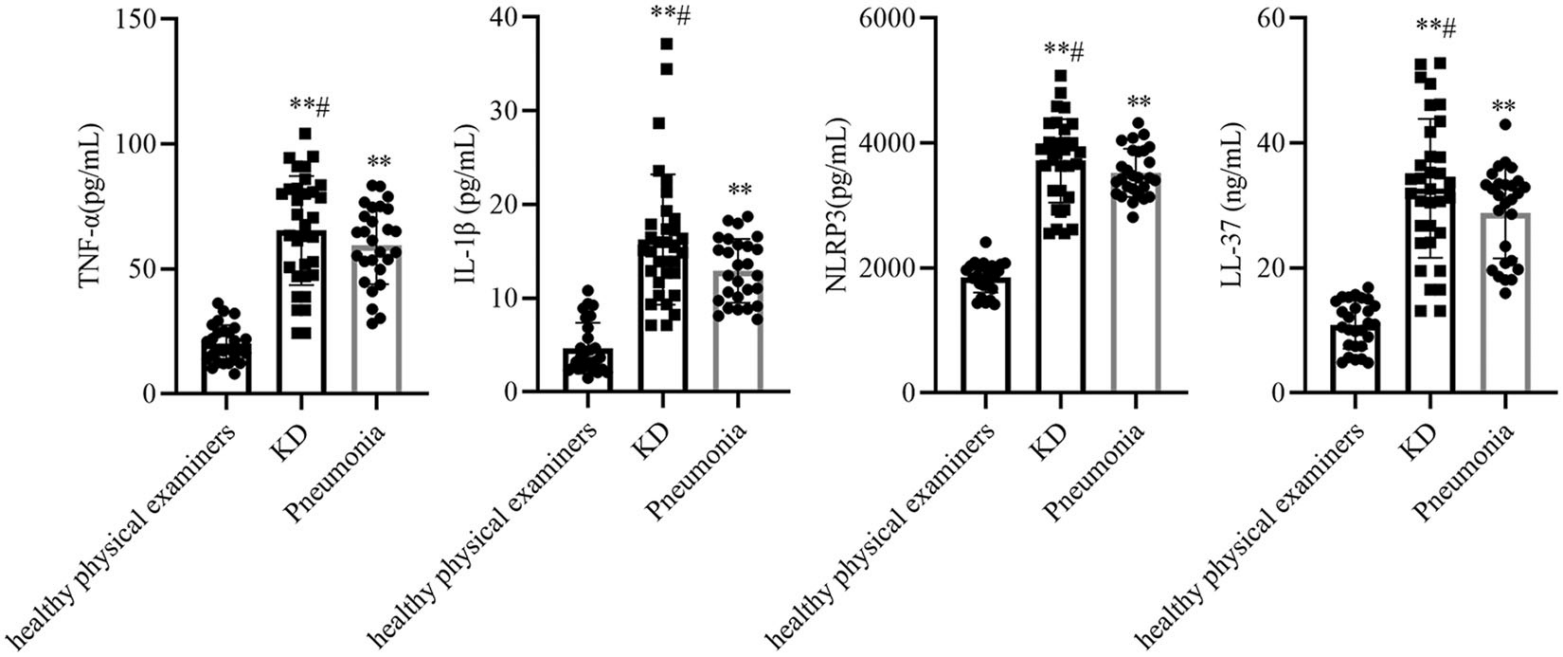
Levels of tumor necrosis factor‐α (TNF‐α), interleukin‐1β (IL‐1β), NLRP3, and cathelicidin (LL‐37) in serum of patients with Kawasaki disease (KD). The levels of TNF‐α, IL‐1β, NLRP3, and LL‐37 in serum. The data are expressed as the mean ± SD. Compared with the healthy physical examiners group, **p < .01. Compared with the pneumonia group, ^#^ *p* < .05.

We collected sera from healthy physical examiners, children with KD, and children with pneumonia and measured the levels of TNF‐α, IL‐1β, NLRP3, and LL‐37 in serum. As shown in Figure 1, the results showed that the levels of TNF‐α, IL‐1β, NLRP3, and LL‐37 in serum were significantly increased in the KD group compared with the healthy physical examiners group (*p* < .01), while the levels of TNF‐α, IL‐1β, NLRP3, and LL‐37 were enhanced in the serum of the KD group compared with the pneumonia group (*p* < .05), indicating the development of a significant inflammatory response in children with KD.

### 3.2 Effects of different concentrations of LL‐37 on the biological behavior and TLR4‐NF‐κB signaling pathway of HCAECs

**Figure 2:**
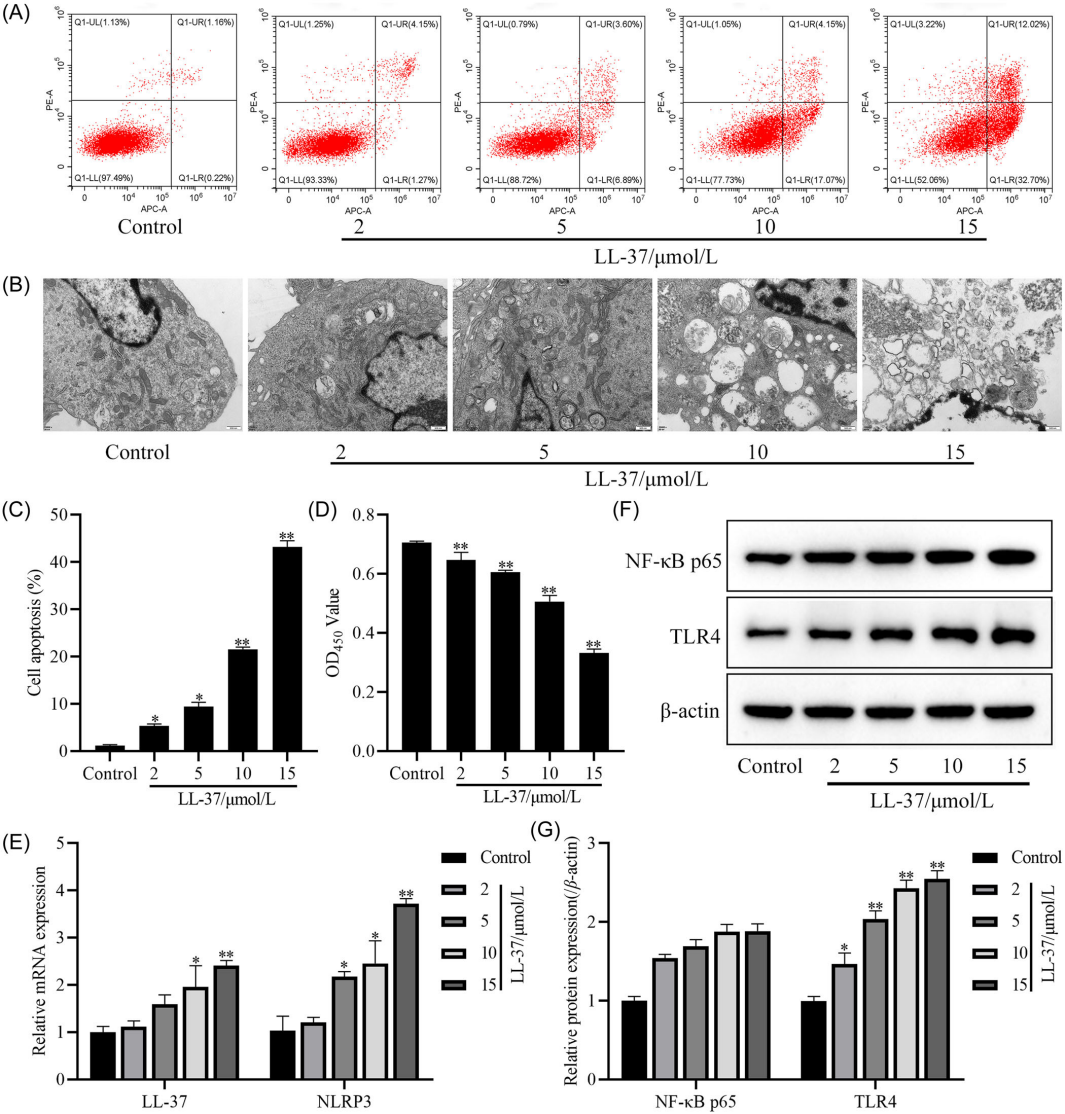
Effects of different concentrations of cathelicidin (LL‐37) on the biological behavior and toll‐like receptors 4 (TLR4)‐NF‐κB signaling pathway of human coronary artery endothelial cells (HCAECs). (A) Flow cytometry scatter plot; (B) transmission electron microscopy observation (25,000×, Scale bar, 500 nm); (C) cell apoptosis; (D) cell absorbance value at 450 nm; (E) the mRNA levels of LL‐37 and NLRP3 in cells; (F, G) the protein expression of NF‐κB p65 and TLR4 in cells was detected by Western blot analysis, those in western blot assays were expressed after being normalized to β‐actin. The data are expressed as the mean ± SD. Compared with the control group, *p < .05 **p < .01.

To analyze the LL‐37‐mediated immunoinflammatory effects and the effects on endothelial cell biology, HCAECs cells were intervened with different concentrations of LL‐37. The results revealed that LL‐37 at 2, 5, 10, and 15 μmol/L promoted apoptosis and inhibited cell proliferation compared with the control group (*p* < .05, Figure 2A,C,D). Transmission electron microscopy showed that the control group had normal cell morphology and structure, and a few autophagic lysosomes and vacuoles were visible in the cytoplasm. The autophagic lysosomes in the cytoplasm of LL‐37 intervention group gradually increased with increasing concentration, and the cell samples underwent necrosis and cytoplasmic lysis after 15 μmol/L of LL‐37 intervention (Figure 2B). In addition, the mRNA expression of LL‐37 and NLRP3 (Figure 2E) and protein expression of TLR4 (Figure 2F,G) were significantly increased in the LL‐37 intervention group at 10 and 15 μmol/L compared with the control group (*p* < .05), indicating that LL‐37 affects the cell biological behavior of HCAECs, promotes the TLR4‐NF‐κB signaling pathway, and had an effect at a concentration of 10 μmol/L. Therefore, subsequent experiments used 10 μmol/L of LL‐37 as the intervention concentration.

### 3.3 LL‐37 stimulates immune inflammatory responses in HCAECs cells by promoting the TLR4‐NF‐κB signaling pathway

**Figure 3:**
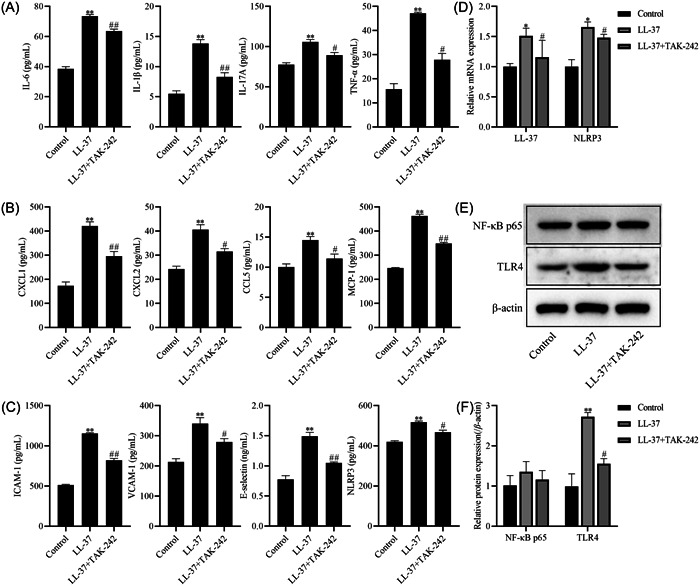
Cathelicidin (LL‐37) stimulates immune inflammatory responses in human coronary artery endothelial cells (HCAECs) cells by promoting the toll‐like receptors 4 (TLR4)‐NF‐κB signaling pathway. (A) The levels of interleukin (IL)‐6, IL‐1β, IL‐17A, and tumor necrosis factor‐α (TNF‐α) in cells; (B) the levels of CXCL1, CXCL2, CCL5, and MCP‐1 in cells; (C) the levels of ICAM‐1, VCAM‐1, ELAM‐1, and NLRP3 in cells; (D) the mRNA levels of LL‐37 and NLRP3 in cells; (E, F) the protein expression of NF‐κB p65 and TLR4 in cells was detected by Western blot analysis, those in western blot assays were expressed after being normalized to β‐actin. The data are expressed as the mean ± SD. Compared with the control group, *p < .05 **p < .01. Compared with the LL‐37 group, ^#^ *p* < .05 ^##^ *p* < .01.

We next demonstrated that LL‐37 stimulates the immunoinflammatory effects of HCAECs cells by promoting the TLR4‐NF‐κB signaling pathway. As shown in Figure 3, Compared with the control group, the levels of inflammatory factors (IL‐6, IL‐1β, IL‐17A, and TNF‐α), chemokines (CXCL1, CXCL2, CCL5, and MCP‐1), and adhesion molecules (ICAM‐1, VCAM‐1, and ELAM‐1) were observably enhanced in the cells of the LL‐37 group, while the levels of the above factors were significantly inhibited by TAK‐242 (*p* < .05, Figure 3A–C). Moreover, the mRNA expression of LL‐37 and NLRP3 (Figure 3D) and protein expression of TLR4 (Figure 3E,F) were significantly reduced in the LL‐37 + TAK‐242 group compared with LL‐37 group (*p* < .05), suggesting that LL‐37 stimulates the immunoinflammatory effects of HCAECs cells by regulating the TLR4‐NF‐kB signaling pathway.

### 3.4 Effect of LL‐37 on the biological behavior of HCAECs cells by modulating the TLR4‐ NF‐κB signaling pathway

**Figure 4:**
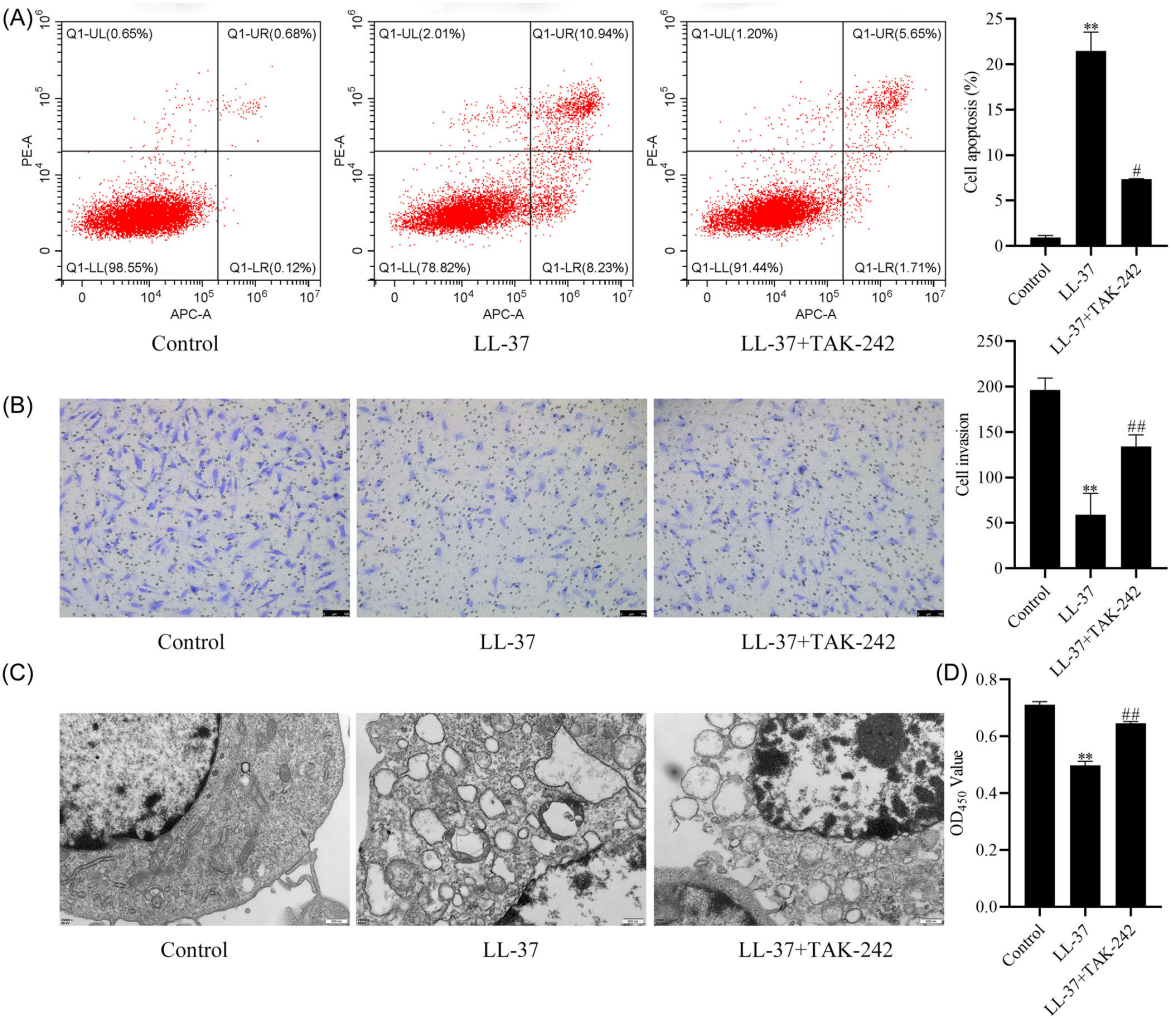
Effect of cathelicidin (LL‐37) on the biological behavior of human coronary artery endothelial cells (HCAECs) cells by modulating the toll‐like receptors 4 (TLR4)‐NF‐κB signaling pathway. (A) Apoptosis by flow cytometry; (B) transwell invasion assay (crystal violet staining, 400×, scale bar, 50 μm); (C) transmission electron microscopy observation (25,000×, Scale bar, 500 nm); (D) cell absorbance value at 450 nm. The data are expressed as the mean ± SD. Compared with the control group, **p < .01. Compared with the LL‐37 group, ^#^ *p* < .05 ^##^ *p* < 0.01.

To determine the effect of LL‐37 on the biological behavior of HCAECs cells through the regulation of the TLR4‐NF‐κB signaling pathway, cell apoptosis, proliferation, cytoskeletal changes, and migration were examined. The results displayed that apoptosis was significantly increased (Figure 4A), cell migration and proliferation were markedly inhibited (*p* < .01, Figure 4B,D), and more autophagic lysosomes were seen in the cell cytoplasm (Figure 4C) in the LL‐37 group compared with the control group. However, the above process was notably reversed by TAK‐242 (*p* < .05), confirming that LL‐37 affects the cell biological behavior of HCAECs by regulating the TLR4‐NF‐κB signaling pathway.

## 4 DISCUSSION

Being one of the most serious hidden dangers affecting children's health, KD has gained more and more attention. Currently, the etiology and pathogenesis of KD are not yet clear. Uncontrolled inflammatory factors are one of the important links in the pathogenesis of KD.^12^ Exploring the possible mechanisms of KD occurrence from the perspective of specific inflammatory factors triggering uncontrolled inflammation will help to explore the important role of inflammatory factor dysregulation in the pathogenesis of KD. In this study, we evaluated the role and mechanism of LL‐37 in the pathogenesis of KD. This study showed that the expression of LL‐37 was significantly elevated in children with KD, and human recombinant LL‐37 promoted apoptosis and inhibited proliferation of HCAECs cells, increased the expression of LL‐37, NLRP3, TLR4 in HCAECs cells, and promoted the level of inflammatory factors, thus participating in coronary artery injury in children with KD.

Studies reported that LL‐37 exhibits modulation of immune and inflammatory factor secretion in inflammatory diseases, such as chronic obstructive pulmonary disease and pneumonia,^21^, ^22^ and neutralizes LPS and thus inhibits TLR4 signaling pathway.^23^ Moreover, LL‐37 can also induce septic shock by activating NLRP3 inflammatory vesicles prompting the production of a series of inflammatory factors such as IL‐1β.^24^ The results of this study showed that serum levels of LL‐37 were elevated in children with KD, implying that the pathological mechanism of KD may be related to LL‐37. HCAECs are an important component of coronary arteries, and apoptosis of HCAECs is an essential factor leading to endothelial dysfunction in coronary arteries. Abnormally activated T cells and monocyte macrophages release a large number of cytokines and inflammatory mediators, which lead to endothelial cell dysfunction, impaired vascular function, lipid deposition, and proliferation of smooth muscle cells, ultimately leading to coronary artery lesions.^25^, ^26^ To further investigate the role of LL‐37, HCAECs were stimulated with human recombinant LL‐37, and as anticipated, LL‐37 significantly promoted apoptosis in HCAECs and significantly increased the expression levels of TLR4 and NLRP3. Previous studies reported that LL‐37 act on endothelial cells and contribute to the production of MCP‐1, ICAM‐1, affect the expression of cytokines IL‐1β, IL‐6, TNF‐a, and induce the production of IL‐17.^27^, ^28^ Our study demonstrated that human recombinant LL‐37 promotes the levels of IL‐6, IL‐1β, IL‐17A, TNF‐α, CXCL1, CXCL2, CCL5, MCP‐1, ICAM‐1, VCAM‐1, and ELAM‐1 in HCAECs, indicating that LL‐37 triggers inflammatory factors to trigger a “waterfall” inflammatory response.

TLR4 can regulate infection‐induced or sterile inflammation by endogenous molecules, and apoptotic process.^29^, ^30^ The TLR4‐NF‐κB pathway is the main regulatory pathway in the NLRP3 initiation phase.^31^ TLR4 activation initiates the Myd88‐dependent signaling pathway to activate the NF‐κB signaling pathway, which in turn initiates the transcription and expression of NLRP3 and IL‐1β precursors, thus triggering a series of inflammatory cascade responses.^32^ Therefore, TLR4‐NF‐κB pathway has an important role for NLRP3 inflammatory vesicles to mediate inflammatory responses. It was found that TNF‐α, IL‐1β, and NLRP3 are all involved in the process of vascular inflammatory injury in KD.^33^ In our study, the serum levels of TNF‐α, IL‐1β, and NLRP3 were significantly elevated in children with KD and pneumonia compared with healthy subjects. Moreover, the elevation of TNF‐α, IL‐1β, and NLRP3 was more pronounced in KD than in children with pneumonia, which may be related to the storm of inflammatory factors triggered by immune imbalance in KD.^12^ In addition, LL‐37 has been found to be associated with inflammation in other diseases and immunity and induced NLRP3 production,^34^, ^35^ suggesting that the uncontrolled inflammatory response may have exacerbated the progression of KD. To further confirm the mechanism of action of LL‐37, the TLR4‐specific inhibitor TAK‐242 was used to intervene in HCAECs, and LL‐37 + TAK‐242 significantly alleviated cellular inflammation and reduced the expression levels of TLR4 and NLRP3 in HCAECs compared with the LL‐37‐treated group, showing that TLR4 signaling contributed to the KD inflammatory response, confirming that LL‐37 could trigger the KD inflammatory response via TLR4‐NF‐κB.

With our present data, we conclude that LL‐37 is highly expressed in children with KD, and its stimulation promotes apoptosis in HCAECs and increases the expression levels of TLR4, NLRP3, and inflammatory factors in the cells, triggering a “waterfall” inflammatory response, which may lead to vascular endothelial cell injury and eventually to coronary artery injury in KD. However, there are some limitations in this study, and whether LL‐37 can regulate other pathways involved in coronary artery injury remains uncertain and needs to be further investigated. In addition, this study was an in vitro experiment, so in vivo studies will need to be continued in the future.

## AUTHOR CONTRIBUTIONS

**Yaheng Lu**: Data curation. **Yizhou Wen**: Data curation. **Tingting Chen**: Formal analysis. **Yingzi Zhang**: Data curation. **Yanfeng Yang**: Project administration.

## CONFLICT OF INTEREST STATEMENT

The authors declare no conflict of interest.

## ETHICS STATEMENT

This study was authorized by the Ethics Committee of the Chengdu Women's and Children's Center Hospital (2022 [35]) and was conducted in accordance with the Declaration of Helsinki.

## Data Availability

The data that support the findings of this study are available from the corresponding author on reasonable request. After the publication of the study findings, the data will be available for others to request. The research team will provide an email address for communication once the data are approved to be shared with others.
